# Stroke patients and visual memory: Exploring the role of spirituality and religiousness

**DOI:** 10.1192/j.eurpsy.2021.1139

**Published:** 2021-08-13

**Authors:** V. Giannouli, K. Giannoulis

**Affiliations:** 1 Institute Of Neurobiology, Bulgarian Academy of Sciences, Sofia, Bulgaria; 2 Faculty Of Theology, Aristotle University of Thessaloniki, Thessaloniki, Greece

**Keywords:** stroke, Spirituality, religiousness, visual memory

## Abstract

**Introduction:**

The relationship of spirituality, religiousness and stroke is a topic of interest.

**Objectives:**

The aim of this preliminary study is to explore whether self-reports in two questionnaires measuring the personal experience of spirituality and religiousness can influence cognition and more specifically performance on neuropsychological tests examining visual memory.

**Methods:**

Fifteen male stroke patients participated voluntarily one year after their hospitalization. The mean age of the patients was 75.58 years (SD = 7.50, range 61-90), level of education 15.47 years (SD = 3.82). In addition to that, fifteen controls with similar demographics, free of physical and mental diseases, were also examined. Depressive symptoms of the participants were assessed with the 15-item Geriatric Depression Scale. The Daily Spiritual Experience Scale, the Systems of Belief Inventory (SBI-15R) and a number of standardized tests examining visual memory were administered: visual perception (copy condition) and memory (Rey-Osterrieth Complex Figure Test-number of correct components on immediate and delayed recall trials and recognition-true positive responses).

**Results:**

indicated a statistically significant difference between the control group and the stroke group in performance regarding visual memory. There was no statistically significant difference between the two groups regarding the levels of spirituality and religiousness.

**Conclusions:**

Visual memory does not seem to be influenced by spirituality and religiousness one year post-stroke. Future research should further investigate the possible influence of the abovementioned factors in post-stroke recovery and rehabilitation.

